# Influence of E-smoking liquids on human periodontal ligament fibroblasts

**DOI:** 10.1186/1746-160X-10-39

**Published:** 2014-09-15

**Authors:** Ines Willershausen, Thomas Wolf, Veronika Weyer, Robert Sader, Shahram Ghanaati, Brita Willershausen

**Affiliations:** 1 Department for Operative Dentistry, University Medical Center of the Johannes Gutenberg University Mainz, Augustusplatz 2, 55131 Mainz, Germany; 2 Institute of Medical Biostatistics, Epidemiology and Informatics (IMBEI), Johannes Gutenberg University Mainz, Mainz, Germany; 3 Department for Oral, Cranio-Maxillofacial and Facial Plastic Surgery, Medical Center of the Goethe University Frankfurt, Frankfurt am Main, Germany; 4 Institute of Pathology, REPAIR-Lab, University Medical Center of the Johannes Gutenberg University Mainz, Mainz, Germany

**Keywords:** Cell proliferation, Electronic cigarettes, Fibroblasts, In vitro, Menthol, Nicotine

## Abstract

**Introduction:**

Over the last years, electronic cigarettes (ECs) have become more popular, particularly in individuals who want to give up smoking tobacco. The aim of the present study was to assess the influence of the different e-smoking liquids on the viability and proliferation of human periodontal ligament fibroblasts.

**Method and materials:**

For this study six test solutions with components from ECs were selected: lime-, hazelnut- and menthol-flavored liquids, nicotine, propylene glycol, and PBS as control group. The fibroblasts were incubated up to 96 h with the different liquids, and cell viability was measured by using the PrestoBlue^®^ reagent, the ATP detection and the migration assay. Fluorescence staining was carried out to visualize cell growth and morphology. Data were statistically analyzed by two-tailed one-way ANOVA.

**Results:**

The cell viability assay showed that the proliferation rates of the cells incubated with nicotine or the various flavored liquids of the e-cigarettes were reduced in comparison to the controls, though not all reductions were statistically significant. After an incubation of 96 h with the menthol-flavored liquid the fibroblasts were statistically significant reduced (p < 0.001). Similar results were found for the detection of ATP in fibroblasts; the incubation with menthol-flavored liquids (p < 0.001) led to a statistically significant reduction. The cell visualization tests confirmed these findings.

**Conclusion:**

Within its limits, the present in vitro study demonstrated that menthol additives of e-smoking have a harmful effect on human periodontal ligament fibroblasts. This might indicate that menthol additives should be avoided for e-cigarettes.

## Introduction

The electronic nicotine-delivery device (electronic cigarettes, ECs) was developed and patented in 2003 by the Ruyan Group (Holdings) Limited in Beijing, China. After their launch on the market in Europe and the United States of America in 2006, e-cigarettes made headlines across the world [1]. Praised as an alternative to conventional cigarettes, e-cigarettes are intended for those who want to reduce or quit smoking. They can be used indoors when restrictions on conventional smoking are present, and have been marketed as environmentally friendly. The e-cigarette is an electronic nicotine delivery device that mimics the outward appearance of a conventional cigarette and has been recently introduced to the market worldwide [2]. While many different models are commercially available, all e-cigarettes consist of little more than three basic components: a battery-part, a heating element and a liquid-containing cartridge. The cartridge can be filled with different e-liquid solutions. These liquids are vaporized by the heating element, and the cigarette is ‘consumed’ by inhaling the liquids’ aerosol mist, thus simulating the act of real tobacco smoking. The e-liquid is a simple solution with usually three main components. They contain diluents added to dilute the mixture and to vaporize upon heating; the most commonly used diluents are propylene glycol and vegetable or aqueous glycerin, usually as a mixture of 80% propylene glycol and 20% glycerin. In addition, flavors are often added to improve the taste of the e-liquid. Nicotine is often added to the propylene glycol in concentrations up to 70 mg/ml. Propylene glycol, a colorless and odorless food additive, is a safe, commonly used chemical without any known harmful effects on human health. Nicotine, in contrast, is considered very harmful to both general and dental health. Tobacco smoke induces changes in cell structure and function by altering cell signaling pathways, which is predominantly harmful to bronchial and oral epithelial cells [3-5]. Adverse consequences on human health have been shown even for low cigarette consumption [6]. Cigarette smoke contains approximately 4800 chemicals, over 60 of which are known to have adverse effects on human tissues and cells, e.g. a high genotoxicity leading to various forms of DNA damage [7]. Tobacco smoking is responsible for 85% of lung carcinomas. According to WHO reports, cancer of the oral cavity is the eleventh most common cancer worldwide [8], while in South Central Asia it is the forth most common cancer [9]. Besides being harmful to overall health, the connection between smoking tobacco and developing periodontal disease is well documented in the literature. Studies have shown that smoking tobacco contributes to the progression of periodontal disease and adversely affects patients’ response to non-surgical or surgical treatment [10-16]. Higher failure rates and increased complications after dental implant placement have also been documented in smokers [17]. Likewise, smoking is a predisposing factor to acute necrotizing ulcerative gingivitis [18]. Exposure to cigarette smoke is also considered an independent periodontal disease risk factor. Although e-cigarettes were initially introduced as a healthy alternative to conventional smoking; however, when they contain e-liquids supplemented with nicotine, health risks associated with conventional tobacco consumption might be expected. In a recent study it was shown that 10 to 81% of the nicotine from the cartridges was vaporized, corresponding to a range of 2.1 to 15.1 mg nicotine [19]. According to a survey among users, e-cigarettes are very popular, because they are supposed to assist with smoking cessation [20]. In in-vitro studies it has been shown that the vapors of the electronic cigarettes were significantly less cytotoxic than cigarette smoke extract [21].

The manufacturing process for e-liquids is not yet standardized, which carries the risk that cancer-inducing substances will enter the wide variety of e-liquids available on the market [22-25]. To date, tobacco-specific substances such as nitrosamines and aldehydes have already been detected in e-liquids. Since no evidence-based studies on the effectiveness of e-cigarettes for smoking cessation presently exist, and since the potentially harmful health effects of e-cigarettes have also not been tested, the use of e-cigarettes to replace cigarette smoking can not be recommended.

The aim of the present in vitro study was to examine, whether e-cigarette liquids and their various flavor components have a damaging influence on the viability and proliferation of human periodontal ligament fibroblast.

## Material and methods

### Cell culture

Clonetics^®^ HPdLF (Human Periodontal Ligament Fibroblasts) were purchased from Lonza (BioWhittaker, Kerviers, Belgium). The fibroblasts were cultured in Dulbecco’s Modified Eagle Medium (Molecular Probes, Carlsbad, CA) containing 1% penicillin/streptomycin (Molecular Probes, Carlsbad, CA) and 10% fetal bovine serum (PAA, Pasching, Austria) and incubated under standard cultivation conditions (37°C, 5% CO_2_ and 95% air). Cells from the 4th to the 6th passage were used for the PrestoBlue Viability Assay, the ApoGlow BioAssay, cell visualization by means of fluorescence staining and the Migration Assay.

### Liquids for electronic cigarettes

The tested e-smoking liquids (eSmokerShop, GmbH, Hannover, Germany) all contained in addition to various flavors the components nicotine (20–22 mg/ml) and, as additive, propylene glycol. Liquids with the flavors hazelnut (filbertone; trans-5-Methyl-2-hepten-4-one; 20 mg/ml nicotine) [26], lime (20 mg/ml nicotine) [27,28] and menthol (5-methyl-2-propan-2-ylcyclohexan-1-ol; 22 mg/ml nicotine) as well as the test substances nicotine and propylene glycol (Sigma-Aldrich Chemie GmbH, Steinsheim, Germany) were included in the investigation. Dose–response curves were established for nicotine and for the menthol-flavored e-smoking liquid. From preliminary experiments with the menthol-flavored liquid a concentration of 10 μg/ml was determined as best suited for all tests described below. To allow for comparisons, all test liquids were therefore diluted with serum-free Dulbecco’s Modified Eagle Medium (DMEM), resulting in a final nicotine concentration of 10 μg nicotine per ml test liquid. There were in total six test groups: control group (PBS); propylene glycol; nicotine; three e-liquids, one with hazelnut flavor, one with lime flavor and one with menthol flavor. The following assays were employed to study the influence of the above mentioned test solutions on the vitality and proliferation of fibroblast cells.

### PrestoBlue Cell Viability Assay

The PrestoBlue Cell proliferation assay (Molecular Probes, Carlsbad, CA) contains a growth indicator which becomes reduced by metabolically active cells to a fluorescent agent. To investigate the influence of the different liquids, the periodontal fibroblasts (10,000 cells/200 μl; n = 6 for each test) were placed into 96 multi well plates and were then incubated up to 96 h with the different liquids. Control cells were incubated only with the same volume of DMEM.

After 0,1,3,6, 24, 72 and 96 h of incubation with 5% PrestoBlue, fluorescence was measured at a wavelength of 530/25 nm and 590/35 nm with a fluorescence reader (Synergy HT-Reader, BioTek, Winooski, VT). Logarithmic signals were converted to a linear scale and expressed as relative fluorescence units (RFU).

### ATP-Detection

The ATP detection was performed with the ApoGlow Bioassay kit (Lonza, BioWhittaker, Kerviers, Belgium). For this assay 30,000 cells/well (n = 6 for each test) were grown to confluence for about 24 h. The supernatants were removed and the cells were incubated for 24 h with the various test liquids. Then ATP was determined using the ApoGlow Bioassay kit. Luminescence was measured with a luminescence reader (Synergy HT-Reader, BioTek, Winooski, VT) and analyzed with Gen 5 (BioTek, Winooski, VT).

### Cell visualization

Calcein-AM/ethidium homodimer II staining (LIVE/DEAD Viability/Cytotoxicity Kit; Molecular Probes, Carlsbad, CA) is a two-color fluorescence-based method for determining viability of cultured cells. Calcein is a fluorogenic esterase substrate that is hydrolyzed intracellularly to a green fluorescent product, which is an indicator of live cells. Ethidium homodimer enters cells with damaged membranes and intercalates with the DNA in the nucleus, emitting a red fluorescent signal. For cell visualization, the fibroblasts were incubated for 24 h with the different test solutions and after removal of the supernatant incubated with calcein-AM (5 μl/10 ml)/ethidium homodimer II (10 μl/10 ml) at 37°C for 15 min. The stained cells were then viewed in an inverted microscope at wave lengths of 450/520 nm (Axiovert 40C, Carl Zeiss, Jena, Germany) at a magnification of ×100.

### Migration assay

The migration assay (Greiner Bio-One GmbH, Frickenhausen, Germany) was performed with Boyden-Chambers (ThinCerts, Greiner Bio-One GmbH, Frickenhausen, Germany). For this investigation the fibroblasts (10,000 cells/well; n = 6 for each test) were placed in Boyden-Chambers. The two compartments of the Boyden-Chamber are separated by a porous PET membrane. Then the test liquids (600 μl with a final concentration of 10 μg/ml nicotine) were added and incubated for 72 h at standard culture conditions. Migratory cells move through the pores. The fibroblasts were stained with calcein-AM (5 μl and 10 ml PBS) for 15 min under culture condition. After incubation the cells were rinsed, placed in a new 24 multi-well chamber, and then incubated for 30 min with 300 μl trypsin/EDTA. Afterwards, aliquots of 200 μl were placed in a black 96 multi-well plate. Fluorescence was measured at a wavelength of 485/20 nm and 520/40 nm with a fluorescence reader (Synergy HT-Reader, BioTek, Winooski, VT) and analyzed with Gen 5 (BioTek, Winooski, VT). Logarithmic signals were converted to a linear scale and expressed as relative fluorescence units (RFU).

### Statistical analysis

The statistical analyses of the data were performed using SPSS 20.0 (SPSS Inc., Chicago, Il.). For descriptive analyses, means and standard deviations were calculated for the normally distributed continuous outcome variables.

For confirmatory analyses two-tailed one-way ANOVA were performed to calculate overall p-values for the comparison of the different groups for each outcome variable.

Normality for outcomes was checked by visual inspection via quantile-quantile plots.

A power analysis for an ANOVA design was performed. With a sample size of n = 6 per group a power between 75% and 100% is available for 7 outcomes.

The global significance level for all statistical test procedures was chosen as α = 0.05.

Post-hoc tests employing the Bonferroni correction for multiple testing were used for comparison of means of two groups.

## Results

**Figure 1:**
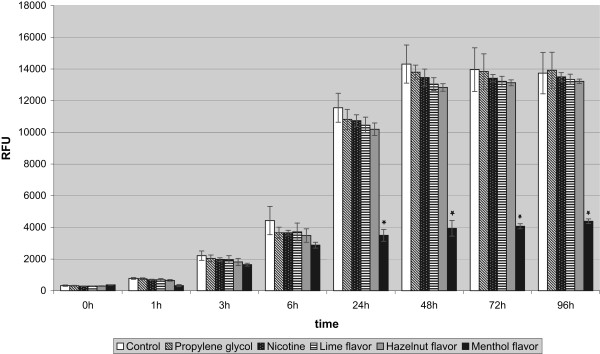
**Effect of the different components and flavors of e-cigarettes up to 96 h, as assessed by PrestoBlue Cell viability assay.** *p < 0.001 for cells incubated with menthol-flavored liquids versus controls for 24 h and for cells incubated with menthol-flavored liquids versus controls and nicotine-treated cells 48-96 h.

As can be seen in Figure 1, the PrestoBlue Cell viability assay showed that over an incubation time of 96 h the proliferation rates of the cells incubated with nicotine or the various flavored liquids of the e-cigarettes were reduced in comparison to that of the untreated control cells, though not all reductions were statistically significant. Starting at 24 h, the highest reduction in the proliferation was observed for the treatment with menthol-flavored liquids, which was the only statistically significant reduction as compared to control cells.

**Figure 2:**
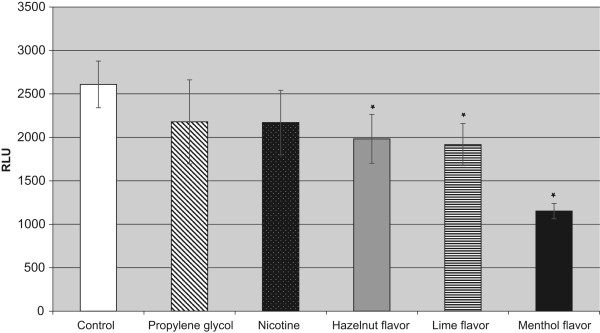
**ATP detection in the fibroblasts at an incubation time of 24 h with the cigarette components and flavors.** *p < 0.024 for cells incubated with hazelnut-flavored liquids versus controls; p < 0.009 for cells incubated with lime-flavored liquids versus controls; p < 0.001 for cells incubated with menthol-flavored liquids versus controls.

**Figure 3:**
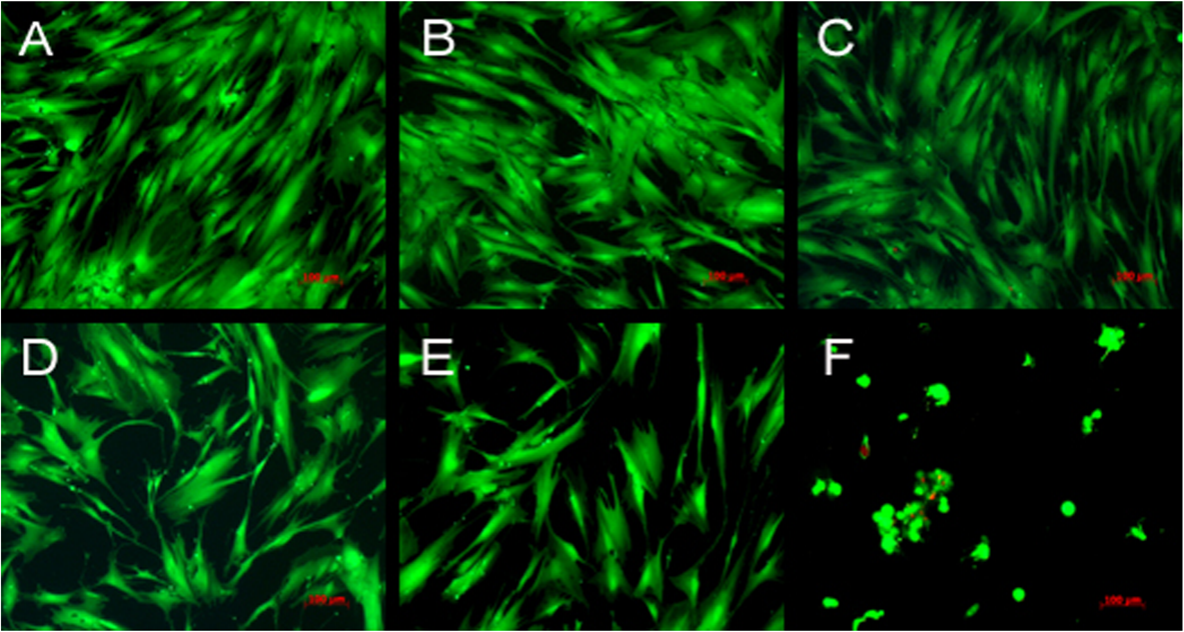
**Reaction of the fibroblasts stained with calcein-AM/ethidium homodimer II.** Reaction of the periodontal ligament fibroblasts to the different liquids (**A** = control; **B** = propylene glycol; **C** = nicotine; **D** = hazelnut flavor, **E** = lime flavor, **F** = menthol flavor), stained with calcein-AM/ethidium homodimer II after an incubation time of 24 h (magnification ×100, bar = 100 μm).

**Figure 4:**
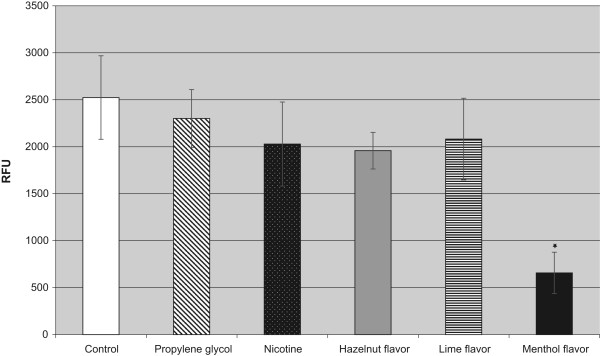
**Effect of the different components and flavors of e-cigarettes on the migration rate of fibroblasts at 72 h.** *p < 0.001 versus controls and nicotine-treated cells.

After an incubation time of 48 h with the menthol-flavored liquid the difference in comparison both to the control cells and the nicotine-treated cells was highly statistically significant (p < 0.001). Hazelnut flavor or lime flavor only caused a slight not statistically significant reduction of the proliferation rates at 48 h. After 96 h of incubation this strong growth-reducing effect of the menthol-flavored liquids persisted and was still statistically significant.More pronounced differences were found using the ApoGlow Bioassay kit for the detection of ATP in fibroblasts after an incubation time of 24 h with the different test substances and flavored liquids (Figure 2). In comparison to the untreated cells, incubation with hazelnut-flavored (p < 0.024), lime-flavored (p < 0.009) or menthol-flavored liquids (p < 0.001) led to a statistically significant reduction of the ATP detection.The findings from the ATP detection assay were confirmed by the visual analysis of the cells stained with calcein-AM/ethidium homodimer II. The untreated human periodontal ligament fibroblasts and those incubated for 24 h with propylene glycol showed good proliferation (Figures 3A,B). Those incubated with nicotine, hazelnut- or lime-flavored liquids showed a slight (Figures 3C-E) growth reduction, while incubation with the menthol-flavored liquid produced a strong growth inhibition (Figures 3F).The inhibitory effect of menthol flavor exposure on the fibroblast cells was especially noticeable in the migration assay (Figure 4). Only the menthol-flavored liquid caused a highly statistically significant reduction (p < 0.001) of cell migration after 72 h in comparison to the control cells as well as to the cells treated with nicotine.

## Discussion

In the present study a possible harmful influence of components and different available flavors of e-cigarettes on human fibroblast cells was examined. E-cigarettes have received a great deal of attention and popularity over the past years and they also play an important role in nicotine replacement therapy [29]. E-cigarettes present a valuable alternative for smokers who are willing to quit. Therefore, it is of interest to what extent the main components of the electronic cigarette smoking liquids are potentially harmful. A novelty in e- cigarettes is flavoring as additive and the liquids come in great variety [24].

In our in vitro study it was demonstrated that some additives from e-cigarettes could cause considerable damage to cell proliferation. While some flavorings such as lime or hazelnut did not seem to exert a harmful effect on cell vitality and proliferation, the menthol e-liquid preparation had significant negative impact on periodontal ligament fibroblasts.

In their in vitro study Semlali et al. [30] explored the influence of whole cigarette smoke on the morphology and function of gingival fiboblasts. The authors were able to prove reduced cellular proliferation and vitality, further a reduction of the number of β1-integrin-positive cells together with an increased LDH activity [26]. These results demonstrate that even a single exposure to cigarette smoke can produce morphological and functional deregulation in gingival fibroblasts.

The effect of numerous refill solutions of e-cigarettes from an American distributor on human embryonic stem cells and human pulmonary fibroblasts were tested, and it was also shown that various additives such as cinnamon or menthol had a cytotoxic potential [31].

It is known that in particular menthol as a tobacco additive has the effect of masking the bitter taste of cigarette smoke; it was first introduced into cigarettes in 1926 [32].

Conventional and electronic cigarettes often contain menthol. Various effects have been suggested for menthol, such as taste ameliorating, cooling, pain-relieving and a slightly numbing effect; menthol ameliorates the cigarette taste, masks the irritating effect of tobacco smoke and makes the smoke easier to inhale [33]. Furthermore, the potential for addiction is increased through its desensitization ability to nicotine, longer exposure and tolerance to nicotine. Finally the risk of cancer is increased by absorption of tobacco smoke components into the lung [33-36]. Menthol can be contained in several parts of cigarettes, such as in the filter, paper and also in flavored liquids.

Recent studies confirm that the addition of menthol leads to a number of negative effects [37,38]. Furthermore, it was shown that menthol increases the flux of the tobacco-related compounds nitrosamine and nitrosonornicotine across porcine esophagus [39]. It is remarkable that one third of diagnosed esophageal cancers in 1998 was found in females [40] and that about a third of female smokers preferred menthol cigarettes [41].

The harmful effect of additives, in particular with menthol flavor could also be observed and confirmed in our study.

Results from numerous studies providing evidence of a negative influence of menthol additives in cigarettes have lead in 2013 to the decision to ban menthol containing cigarettes in the European Community; in addition, it was proposed to harmonize the regulations of e-cigarettes, which presently differ between the countries in the EU, and to restrict their advertising [42]. Since the present study shows that in particular menthol additives in e-cigarettes had a harmful effect on human fibroblast cells, it might be considered to prohibit the use of this additive not only in conventional cigarettes but also in e-cigarettes.

Within its limits, the present in vitro study demonstrated that menthol additives of e-smoking have a harmful effect on human periodontal ligament fibroblasts. This might indicate that it is preferable for e-cigarettes not to contain menthol additives.

## Competing interests

The authors declare that they have no competing interests.

## Authors’ contributions

IW, TW and BW carried out the study. IW and VW performed the statistical analysis. IW, TW, BW , RS and SG conceived of the study, and participated in its design and coordination. All authors read and approved the final manuscript.
